# Elastin-Coated Biodegradable Photopolymer Scaffolds for Tissue Engineering Applications

**DOI:** 10.1155/2014/624645

**Published:** 2014-10-23

**Authors:** Rossella Barenghi, Szabolcs Beke, Ilaria Romano, Paola Gavazzo, Balázs Farkas, Massimo Vassalli, Fernando Brandi, Silvia Scaglione

**Affiliations:** ^1^National Research Council of Italy (CNR), IEIIT Institute, Via De Marini 6, 16149 Genova, Italy; ^2^Department of Nanophysics, Istituto Italiano di Tecnologia (IIT), Via Morego 30, 16163 Genova, Italy; ^3^National Research Council of Italy (CNR), IBF Institute, Via De Marini 6, 16149 Genova, Italy; ^4^National Research Council of Italy (CNR), INO Institute, Via Moruzzi 1, 56124 Pisa, Italy

## Abstract

One of the main open issues in modern vascular surgery is the nonbiodegradability of implants used for stent interventions, which can lead to small caliber-related thrombosis and neointimal hyperplasia. Some new, resorbable polymeric materials have been proposed to substitute traditional stainless-steel stents, but so far they were affected by poor mechanical properties and low biocompatibility. In this respect, a new material, polypropylene fumarate (PPF), may be considered as a promising candidate to implement the development of next generation stents, due to its complete biodegradability, and excellent mechanical properties and the ease to be precisely patterned. Besides all these benefits, PPF has not been tested yet for vascular prosthesis, mainly because it proved to be almost inert, while the ability to elicit a specific biological function would be of paramount importance in such critical surgery applications. Here, we propose a biomimetic functionalization process, aimed at obtaining specific bioactivation and thus improved cell-polymer interaction. Porous PPF-based scaffolds produced by deep-UV photocuring were coated by elastin and the functionalized scaffolds were extensively characterized, revealing a stable bound between the protein and the polymer surface. Both 3T3 and HUVEC cell lines were used for *in vitro* tests displaying an enhancement of cells adhesion and proliferation on the functionalized scaffolds.

## 1. Introduction 

Stent angioplasty is a revolutionary technique introduced in 1980 and widely used in the past years to treat the atherosclerosis [1]. Currently available stents are tiny, expandable metallic mesh tubes, typically made of medical-grade stainless steel or cobalt alloy. Once implanted, the stents are placed* in situ *and expanded through inflated balloon; thus, they remain permanently in the artery [1]. Despite the good mechanical properties of these materials and the initial success of such implants, in clinical practice a new artery occlusion in up to 18% of cases within 2 weeks of implantation has been observed, mostly related to foreign body immune response reactions [1]. In particular, to isolate the stent from blood, the organism reacts with a platelet proliferation and with smooth muscular cells migration on the metal surface and their hyperproliferation, finally causing thrombosis, neointimal hyperplasia, and in-stent restenosis [1–3].

To solve these problems, in the last years, a new generation of stents, namely, the drug eluting stents, has been proposed [4]. These bioactive stents loaded with specific antiproliferative drugs have displayed impressive clinical benefits concerning the inhibition of neointimal cell proliferation. However, their use has highlighted some safety concerns related to stent thrombosis: the presence of these antiproliferative drugs prevents the isolation of metal from blood causing a continuous exposure of a free metallic surface and, accordingly, a nonstop immune response by the organism [1, 5].

The need to unravel these clinical concerns, besides the unresorbability of such metallic stents, which remain permanently* in situ*, has fostered numerous research groups to explore new biodegradable and bioactive polymers being able to be gradually resorbed by the body, reducing thrombosis and neointimal hyperplasia [2]. Some novel stent coating strategies have already been proposed [2, 3, 6–8]; however, these approaches are still at the preliminary stage and their long-term behavior is still unknown [3].

In this scenario, relevant developments in the field of tissue engineering have recently emerged by exploiting a biomimetic approach in which biomaterials are designed to replace the internal lining of blood vessels [2]. In particular, engineered polymeric scaffolds have been functionalized to incorporate naturally inspired molecules, being able to induce specific cellular responses and guide tissue regeneration [9]. In this context, the adoption of elastin-based materials has raised a strong attention by mimicking many features of the extracellular matrix with a potential to promote the migration, growth, and organization of cells during regeneration processes [10–12]. Moreover, the efficiency of such approach for vascular applications was also demonstrated, showing the ability of elastin-based materials to interact and favour the growth of endothelial cells [3, 13] to inhibit smooth muscle cells proliferation [14] and to be antithrombogenic [15, 16].

The effectiveness of elastin-based materials in cardiovascular tissue regeneration has suggested the adoption of biodegradable scaffolds as substrates for advanced stent fabrication. For this reason, we have used a biodegradable, cytocompatible, and photocrosslinkable polymer, poly(propylene fumarate) (PPF), to build and shape scaffolds in a standardized manner through mask-projection photocuring with excimer laser. This technique received considerable attention due to its simplicity, rapidity, and scalability, together with a resolution suitable to prepare 3D microstructures for tissue engineering applications [17]. Basically, both the microarchitecture and the macroshape of the produced rigid constructs, called scaffolds, can be controlled by applying the proper mask upon excimer laser exposure. The pore dimensions can be tuned to better accommodate the elastin fiber and enhance the adhesion and stability of the coating by means of morphological cues. As for the perspective of biodegradable stents, the mask-projection excimer laser stereolithography allows the fabrication through the layer-by-layer approach of 3D scaffolds (i.e., stents) not only with the proper macrogeometry (diameter, length, and meshed shape), but also with the optimal morphology on the microscale. This allows achieving a better and more stable elastin coating by tuning the pore and groove dimensions on the micrometer scale [18]. In general, diethyl fumarate (DEF, the monomer used for the synthesis of PPF) is blended to the polymer to reduce the viscosity that is necessary for the production.

In a previous work, sterilization methods of excimer laser photocured PPF : DEF scaffolds and their degradation profile were investigated for a possible use in clinical applications [19]. In this study, we report on the effect of elastin coating on such PPF : DEF scaffolds in order to improve fibroblast and endothelial cell adhesion and proliferation since our assumption has been that, through a biomimetic approach, the elastin functionalization of porous PPF : DEF substrates may improve the cell-polymer interaction.

Elastin-coated PPF : DEF scaffold prototype was realized and extensively characterized. The long-term stability of the coating, protein fibres assembly, mechanical performances, and* in vitro* cellular interaction were also investigated.

## 2. Materials and Methods

### 2.1. Preparation of Polypropylene Fumarate Resin

PPF : DEF was synthesized as reported in detail in [20]. Briefly, PPF : DEF was prepared by a condensation reaction between fumaric acid and propylene glycol with a molar ratio of 0.8. In a triple-neck flask with an overhead mechanical stirrer, a thermometer, and a Barrette trap connected beneath the condenser, the reaction was conducted in 140°C for 16-17 hours and then in 180–190°C for 4-5 hours. During the first hours of the reaction, water was collected as a byproduct; then, with an increasing temperature the unreacted propylene glycol and low molecular weight impurities were removed. After keeping the product at room temperature overnight, it was purified by rotary evaporation in CH_2_Cl_2_. Finally, PPF was blended with DEF in ratio 7 : 3.

### 2.2. Elastin Coating: Effect of pH and Temperature

Elastin powder from bovine neck ligament, purchased from Sigma-Aldrich (code E1625), was suspended in a buffer solution (Tris 0.2 M in PBS) under gentle stirring to obtain a concentration of 20 mg/mL. 50 *μ*L of the suspension was statically placed on each scaffold. The remaining solution and powder were stored at 4°C. In order to test the stability of elastin coating, different pH and working temperatures were tried, as reported in Table 1. The buffer pH was modulated by adding 0.2 M of HCl, to obtain three values: 8.3, 8.8, and 9.3. Then elastin was suspended in the buffers and deposited on PPF : DEF scaffolds; glass substrates were used as control. Samples were finally incubated at 24°C, 37°C, and 50°C for 72 hours, for a total of 9 different incubating conditions for each substrate (Table 1). After 72 hours, samples were analysed by scanning electron microscopy [21], fluorescence, and transmission optical microscopies to evaluate the fiber adhesion, morphology, and packing on various substrates. Samples were then incubated for 24 hours in distilled water at 37°C, dried, and analysed again to evaluate the elastin adhesion and stability.

### 2.3. Fourier Transform Infrared Spectroscopy (FTIR)

FTIR analysis was performed to evaluate the elastin adsorption on the PPF : DEF scaffolds and the eventual presence of chemical bonds between elastin and PPF : DEF. FTIR spectra were collected at the resolution of 0.5 cm^−1^ and signal average of 16 scans in each interferogram over the range 4000–500 cm^−1^ using a FTIR Spectrum Two spectrometer. Pure PPF : DEF scaffolds were analysed as negative control. Variations in the spectral data were assessed by comparing the relative changes in peak intensities of the IR-absorbing functional groups.

### 2.4. Elastin Fibres Packing Measurements

Elastin in Tris buffer was deposited separately on 10 glass substrates, incubated for 72 hours at 37°C, and then analysed by scanning electronic microscope [21] to evaluate the protein fibres assembly. From the acquired images, the numbers of fibres per area and fibres bundles diameter and length were derived with a manual procedure implemented through an open source image processing software [22]. Statistical analysis was then performed and the mean values and standard deviations were derived.

### 2.5. *In Vitro* Cell Culture

NIH 3T3 mouse embryonic fibroblasts were expanded in D-MEM supplemented with 10% fetal calf serum and 1% penicillin/streptomycin. When the required confluence was reached, cells were detached with 0.05% trypsin, counted, and seeded onto the surface of scaffolds, at a density of 5 × 10^4^ cells/cm^2^ and a concentration of 0.5 × 10^6^ cells/mL. All scaffolds were previously irradiated for 72 hours at 5000 rad of gamma ray to obtain high sterility. Samples were cultured up to 3 weeks in an incubator at 37°C in an atmosphere of 5% CO_2_ to allow gas exchange. The medium was changed twice a week.

After 72 hours of culture, scaffolds were washed with phosphate-buffered saline (PBS), fixed with 4% paraformaldehyde, and processed to investigate the cellular adhesion on the substrates.

After 1 and 3 weeks of culture, samples were analysed to evaluate long-term cellular proliferation. All experiments were performed in duplicate.

Human umbilical vein endothelial cells (HUVEC) were expanded in a specific low serum endothelial cell medium (C-22110 Promocell Heidelberg, Germany) supplemented with 1% penicillin/streptomycin. After reaching confluence, cells were detached and seeded following the same procedure. Samples were harvested 24 hours after cell seeding and cellular adhesion was evaluated.

### 2.6. Scanning Electron Microscopy [21]

The scaffolds were dehydrated in ethanol solutions at 70%, 80%, 90%, and 100% for 5 min each and then dried in air. Finally, they were fixed on aluminium stubs and sputter-coated with a 10 nm thick gold layer. SEM images were acquired at 15 kV by using a JSM-6490 scanning electron microscope (JEOL, Japan) and processed to analyse the scaffolds geometry, elastin coating, cell adhesion, morphology, and proliferation.

### 2.7. Cells Viability

To determine cellular viability within the scaffolds, a live/dead assay (Live/Dead Cell Double Staining Kit, Sigma-Aldrich, Italy, code 04511) was performed after 3, 7, and 21 days. The elastin-coated scaffold samples were incubated with the live/dead staining solution at 37°C for 15 min and then imaged by using an upright microscope equipped with transmitted illumination and epifluorescence (Eclipse Ni-U, Nikon, Japan). For each sample, six acquired images were processed, in duplicate, for each time point, using open source image analysis software [22].

Live cells (calcein AM stained—green) and dead cells (propidium iodide stained—red) were measured. Statistical analysis was then performed and the results are presented as mean ± standard deviation.

## 3. Results

### 3.1. PPF : DEF Scaffolds Fabrication

Two-dimensional scaffolds were obtained using the mask-projection excimer laser photocuring setup presented in [19]. The light source was a XeCl excimer laser (CompexPro 110) operating at 308 nm with laser pulse duration of 20 ns and repetition rate up to 100 Hz. The mask image was projected on the target using a demagnification of 4. The numerical aperture of the projection lens was about 0.1 and the laser pulse fluence was controlled by means of a variable attenuator.

A photoinitiator (BAPO) is added to the polymer resin to enable the photocrosslinking reaction upon the exposure of UV irradiation [23–25]. Changing the parameters used for photocuring, such as photoinitiator and/or monomer concentration, and laser wavelength, pulse repetition rate, and pulse fluence (energy density per unit area), it is possible to achieve different aspect ratios and Young modulus of the produced scaffolds [24]. Finally, by using different masks, it is possible to produce a large variety of structures with desired porosities and to fabricate scaffolds, which are the most suitable for targeted applications in tissue engineering.

**Figure 1:**
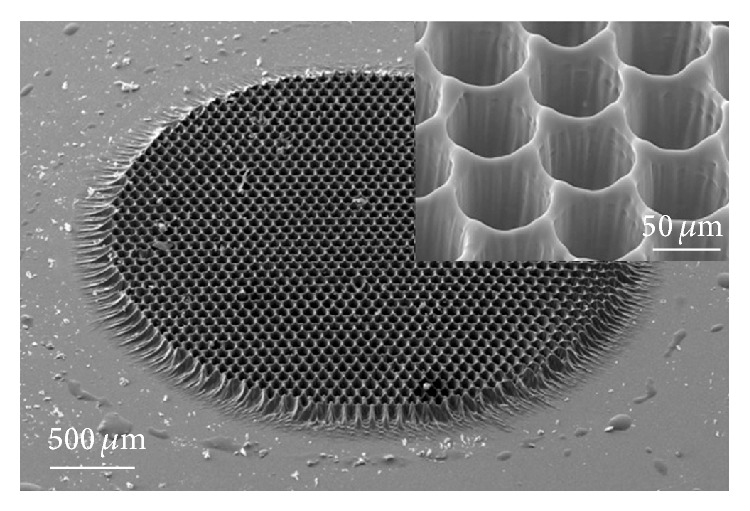
Tilted SEM view of a 2D scaffold fabricated by the proposed method. The inset shows the pores in a higher resolution.

Scaffolds with round-shaped pores were produced using PPF : DEF resin with 1% BAPO and applying 264 laser pulses. The size of each circular metallic spot on the mask is 220 *μ*m and the spacing between them is 80 *μ*m in all directions, which, due to the 4-time demagnification, results in a pore diameter of 55 *μ*m in diameter and 20 *μ*m spacing between each pore. In Figure 1 a reference SEM image of a scaffold (3 mm in diameter) produced by following this protocol is shown.

### 3.2. Elastin Functionalization and Morphological Characterization of Scaffolds

**Figure 2:**
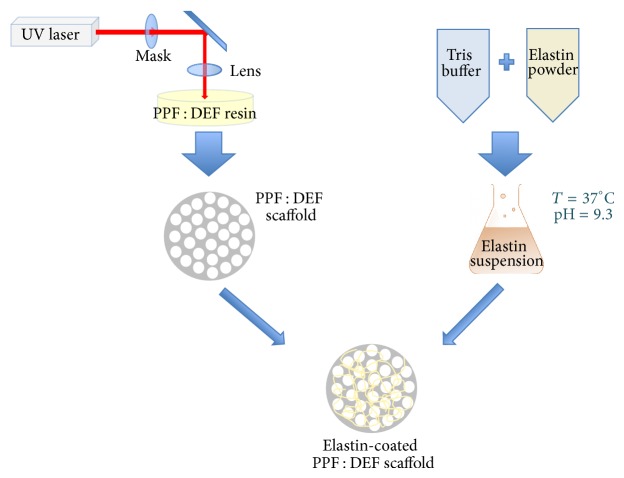
Schematic of elastin-coated PPF : DEF scaffold preparation.

Elastin coating on the PPF : DEF substrates was performed as schematically shown in Figure 2. Different pH-temperature conditions were tested and qualitatively evaluated under fluorescence microscopy exploiting the autofluorescence of the protein. The analysis was carried out both prior to and after incubation in physiologic buffer to verify the stability of the coating and finally choose the most proper experimental conditions (i.e., buffer pH and incubation temperature). It was observed that a homogeneous and spatially uniform distribution of elastin fibres was obtained when working at high pH (9.3) buffer and at 24°C (incubation time: 72 h).

To test the stability of the functionalizing coating, the scaffolds were incubated for 24 h at 37°C in physiologic buffer and then analysed by fluorescence microscopy and FTIR. Interestingly, the elastin-coated PPF : DEF scaffolds did not show any significant difference compared to those analysed before incubation in liquid, indicating a strong stability of the coating and suggesting the onset of a specific bond between the elastin and polymeric substrate.

**Figure 3:**
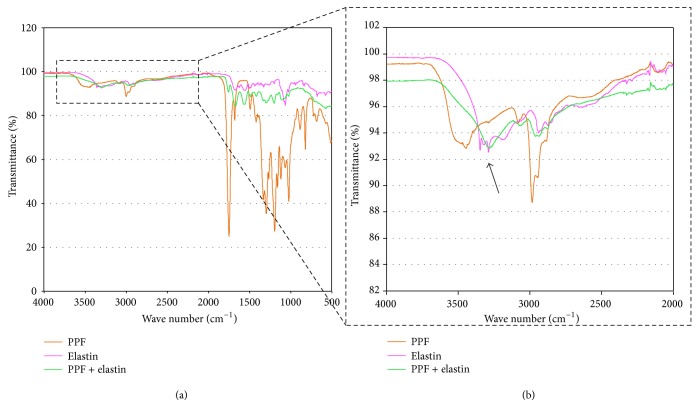
FTIR analysis ((a) and (b)) of the elastin powder (pink line), PPF : DEF scaffold functionalized with elastin (green line) and PPF:DEF scaffold (orange line) after 24 h of incubation in distilled water at 37°C. The arrow indicates a change of the transmission percentage in the range of wave number (3500–3000 cm^−1^) related to the OH extremities of the PPF : DEF and amidic group in elastin. (b) shows spectrum in the range of wave number (4000–2000 cm^−1^) at higher resolution.

To assess this aspect, FTIR analysis was performed (Figure 3), showing a change in the range 3500–3000 cm^−1^, corresponding to the onset of an O–H bond in the functionalization process, with respect to bare PPF : DEF (Figure 3(b), blue line). This measurement highlights the formation of a new chemical bond associated with the reaction of the elastin amide group with free OH extremities of the polymer. Differently, in the range 2000–1000 cm^−1^, the background contribution of PPF : DEF is too high to appreciate small changes supposedly induced by the presence of new bonds.

**Figure 4:**
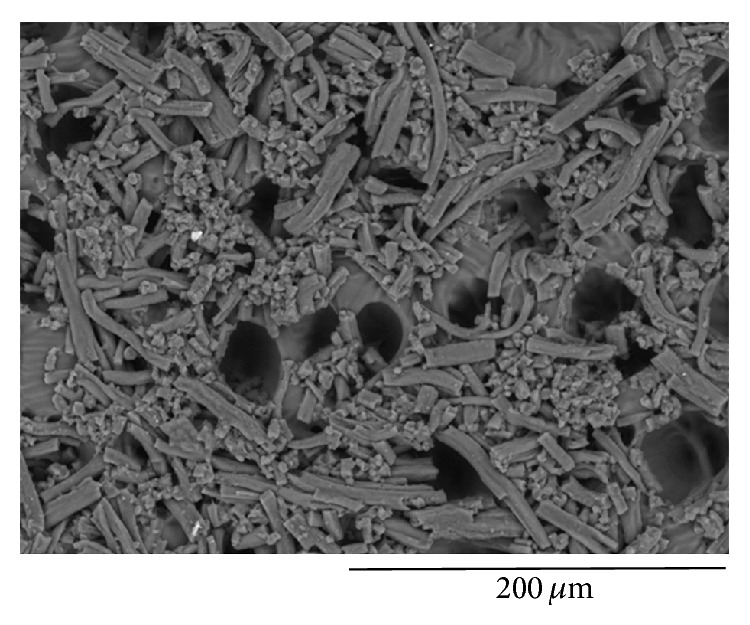
SEM analysis of elastin fibers homogeneously covering the PPF : DEF scaffold.

In terms of morphology, we noticed the tendency of elastin to aggregate and form fibrillar self-organized structures (Figure 4). Fairly long fibres with a mean diameter of (6.53 ± 2.30) *μ*m were observed to build bundles of fibrils (packing). The ratio (the length of these aggregates *L* divided by their total diameter *D*) was found to be quite constant (*L*/*D* = 3.82 ± 0.90).

### 3.3. Cells Culture

To evaluate the cell-substrate interaction, PPF : DEF scaffolds, either functionalized or not with elastin, were cultured with cells up to 3 weeks; SEM analysis and live/dead assay were performed at different time points.

After 3 days of culture, we observed 3T3 fibroblast cells adhering to the polymeric mesh. No significant difference was noticed between the elastin-coated and noncoated PPF : DEF scaffolds.

**Figure 5:**
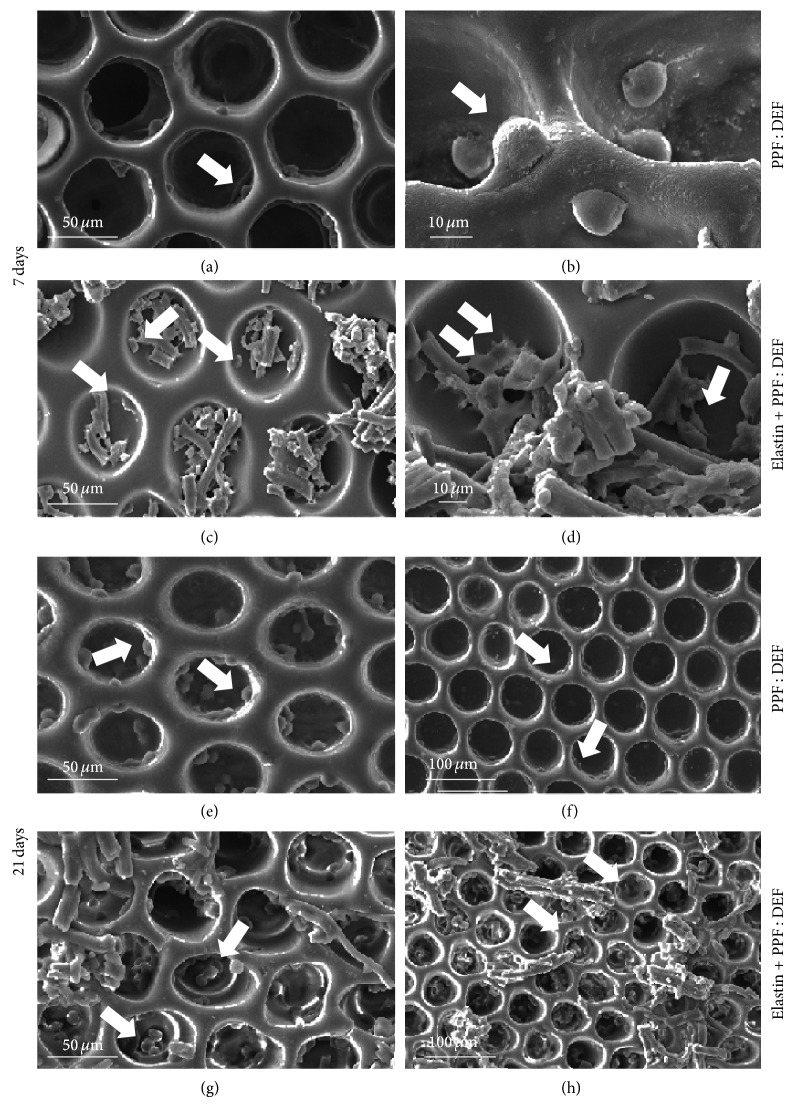
SEM images showing 3T3 cell culture after 7 days of culture on PPF : DEF scaffolds without elastin ((a), (b)) and coated with elastin ((c), (d)), and after 21 days of culture on PPF : DEF without elastin ((e), (f)) and with elastin ((g), (h)). Arrows highlight cells.

After a longer cell culture time (7 days), cells were well adhering to PPF : DEF (Figure 5, panels (a)-(b)) and functionalized substrates (Figure 5, panels (c)-(d)), showing a tendency to populate the inner walls of the scaffold pores. In particular, for elastin-coated scaffolds, it was possible to observe cells directly interacting with elastin fibers (see arrows); moreover, cells were more spread compared to those observed on bare PPF : DEF.

This behavior was confirmed also after 21 days of culture (Figure 5, panels (e)–(h)) in which an increase of the number of cells colonizing the substrates was observed, strongly more pronounced for elastin functionalized substrates (panels (g)-(h)).

**Figure 6:**
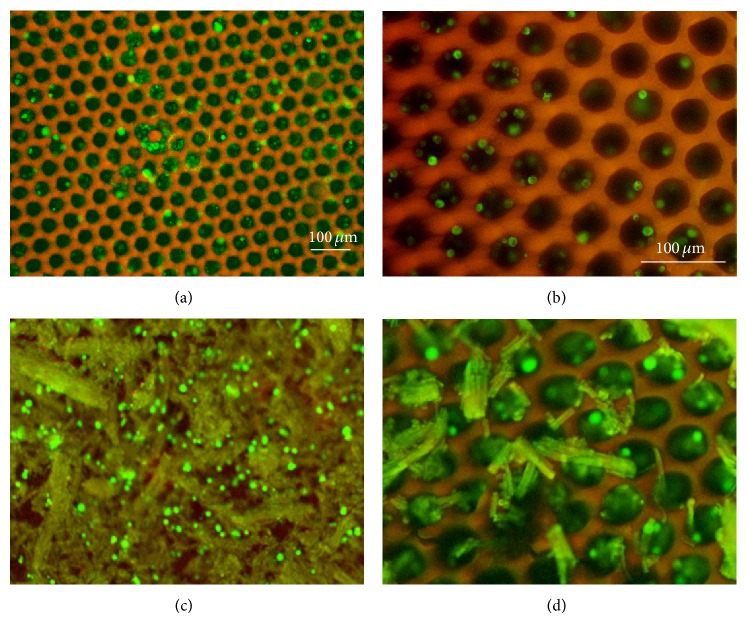
Fluorescence microscopy images showing live 3T3 cells (green) and dead cells (red) after 7 days of culture on PPF : DEF scaffolds without elastin ((a), (b)) and coated with elastin ((c), (d)), at low magnifications ((a), (c)) and high magnifications ((b), (d)). Scale bar: 100 mm.

The live/dead assay was performed to evaluate 3T3 cells viability on the elastin-coated scaffolds (Figure 6): after 7 days of culture, uncoated PPF : DEF scaffolds displayed a very small number of cells colonizing them compared to the functionalized ones (panels (a)-(b) and (c)-(d), resp.), providing an evidence for the bioactive function of the elastin coating (560 cells/mm^2^ versus 430 cells/mm^2^). Interestingly, in both cases, few cells died, marked with red spots, during the culture (Figure 6).

**Figure 7:**
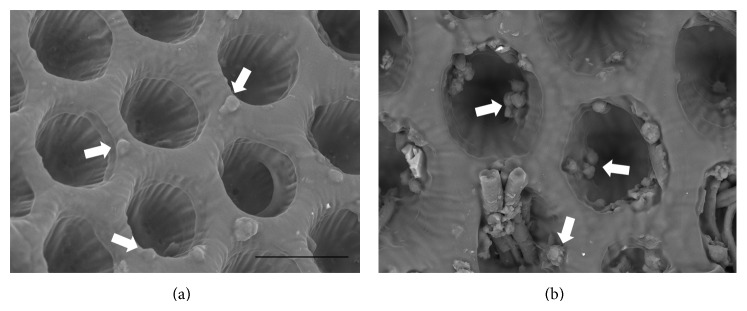
SEM images of HUVECs after 1 day of culture. Arrows indicate cells adhering on the substrate. Scale bar: 50 mm.

Besides the 3T3 cell culture, the elastin-coated PPF : DEF scaffolds were also tested* in vitro *by culturing endothelial cells (HUVEC cell line) on them. SEM images (Figure 7) show a notable proliferation of cells on elastin-coated scaffolds (Figure 7(b)) compared to the bare PPF : DEF (Figure 7(a)).

## 4. Discussion

One of the key features of a material for advanced stent fabrication is the ability to be eliminated or resorbed by the organism once its stabilizing function is completed. In fact, the mechanical function of the stent, to keep open a failing vessel, is accomplished in about one year during which the organism has time to recover its autonomous functionality. Therefore, the use of resorbable polymers as a base material has been widely encouraged.

In a past work, we have investigated the resorption behaviour of PPF : DEF polymer, providing a fully quantification of its resorbability, with a structure's mass decrease corresponding to a halving time of about one year [19]. Based on its resorbable behavior and excellent biomechanical performance [26], we have thus considered such material as a good candidate for scaffolding applications in vascular surgery applications. In particular, in this work, we aim to identify and successfully verify a functionalization strategy for PPF : DEF porous scaffolds to enhance their potential for advanced stent design and production. The possibility to microfabricate controlled structures of PPF : DEF by means of mask-projection excimer laser photocuring technique was previously presented [24]. We here have demonstrated that elastin coating allows establishing a stable and bioactive functionalization capable of promoting and fostering cellular activity* in vitro*.

A controlled porous architecture has been designed and realized by applying mask-projection excimer laser photocuring techniques. The pore dimension was tuned to accommodate the elastin coating and enhance the adhesion and stability of the coating by means of morphological cues. In the perspective of biodegradable 3D stent, the mask-projection excimer laser stereolithography allows not only fabricating stents with the proper macrogeometry (diameter, length/height, and meshed shape), but also controlling the morphology on the microscale. The selection of appropriate scaffold architecture with suitable pore mesh has long been recognized as the key factor that may influence the mechanical strength and determine tissue remodelling [18].

However, being chemically inert, PPF is not able to elicit any biological reaction or even promote tissue regeneration or material integration. A biomimetic strategy was thus undertaken to activate the scaffold by means of elastin coating. This protein is ubiquitously present in human body; moreover, it constitutes the main component of the innermost layer of blood vessels, the tunica intima. It is therefore expected that elastin-coated materials can play a relevant role in activating the correct response in the injured vessel. This biological function was already demonstrated in several studies [2, 15, 24]. In addition, it was reported that elastin promotes* in vivo* endothelial cells adhesion and proliferation [27], which are responsible for all those mechanisms that regulate the platelet and smooth muscular cells overgrowth [28]. In particular, elastin displays a natural antithrombotic effect, which prevents the new obstruction of blood vessels [2, 18, 29].

Modulating the experimental parameters for elastin deposition, a stable procedure was achieved, allowing elastin to link on PPF : DEF scaffolds with a suitable stability. In particular, FTIR analysis showed that the interaction between elastin and the substrate is mediated by the formation of a covalent bond exploiting amine residues of elastin. After incubation in water, these new bonds remained unchanged allowing supporting cell culture for long periods.

Besides chemical-structural characterization, functionalized PPF : DEF prototypes have also been biologically tested* in vitro* with fibroblast and with endothelial cells.

Fibroblasts were cultured for both short and long terms to evaluate cellular adhesion and proliferation onto pure and elastin functionalized PPF : DEF substrates. An important evidence was the direct interaction between cells and elastin coating, as highlighted by the SEM analysis (particularly visible in Figure 6), while the absence of spreading cells was observed on the bare PPF : DEF scaffolds. Only few dead cells were detected in both cases, which confirms that the scaffold is not chemically cytotoxic. However, without functionalization, cells remained round-shaped, even after 21 days. Interestingly, elastin coating allowed also the fibroblast proliferation to enhance over time, as confirmed by live/dead fluorescence cell analysis. These latter results were in accordance with a previous work showing that tropoelastin, a precursor of elastin, promotes the fibroblast adhesion, proliferation, and spreading [30].

Besides the fibroblast cell culture, also endothelial cells were cultured on the elastin-coated scaffolds. Some groups demonstrated that elastin-like polypeptide and tropoelastin enhanced endothelial cells adhesion [31], finally forming a new endothelial monolayer [3]. However, Williamson et al. reported that endothelial cells poorly attached to elastin substrates compared to other cellular lines [32]. According to these data, our results highlight an enhanced survival and adhesion of HUVECs on the functionalized substrates after 24 hours, confirming the positive effect of elastin functionalization. Nevertheless, at a prolonged time, endothelial cells were found to poorly adhere to the substrates [2, 31]. Endothelial cells are difficult to culture alone for up to 7 days because of their needs of particular additives, such as growth factors or controlled glucose levels [33, 34].

The adhesion of endothelial cells to functionalized substrates demonstrates the possibility to bioactivate the polymeric graft for vascular applications. PPF : DEF material was selected among several polymers on the basis of its tunable mechanical properties and degradability [19]. The suitability of PPF : DEF as a novel vascular material was here demonstrated by enhancing its cellular affinity through a biomimetic surface functionalization.

## 5. Conclusions

The innovative, functionalized scaffold obtained by means of microfabricated biodegradable polymer structures coated with elastin is an interesting platform to design new materials and tools to be utilized in vascular surgery and regenerative medicine. A biomimetic strategy developed by using elastin coating on biodegradable photopolymer scaffolds showed worthy results in terms of cells adhesion and proliferation. These results have a foundational character for the use of PPF : DEF scaffolds in vascular tissue engineering applications and open further experimentation towards* in vivo* applications. In particular, a detailed study of the optimal construction and functionalization parameters (scaffold morphology and rigidity, elastin management) for the reduction of multipotential stromal cells hyperproliferation in a coculture of fibroblast, smooth muscle cells, and endothelial cells would putatively provide a strong candidate for innovative vascular prostheses.

## Figures and Tables

**Table 1:** Incubation conditions of elastin.

*T*/pH	8.3	8.8	9.3
24°C	Glass/PPF : DEF	Glass/PPF : DEF	Glass/PPF : DEF
37°C	Glass/PPF : DEF	Glass/PPF : DEF	Glass/PPF : DEF
50°C	Glass/PPF : DEF	Glass/PPF : DEF	Glass/PPF : DEF
